# Retrospective Analysis of Cardiovascular Effects of FES Cycling in People with Complete and Incomplete Spinal Cord Injury

**DOI:** 10.3390/jcm15051967

**Published:** 2026-03-04

**Authors:** Mariann Mravcsik, Amelita Fodor, Balazs Radeleczki, Melinda Feher, Peter Cserhati, Andras Klauber, Jozsef Laczko, Lilla Botzheim

**Affiliations:** 1 Department of Computational Sciences, HUN-REN Wigner Research Centre for Physics, Konkoly-Thege Miklos Street 29-33, 1121 Budapest, Hungary; fodor.amelita@wigner.hun-ren.hu (A.F.); radeleczki.balazs@wigner.hun-ren.hu (B.R.); laczko.jozsef@wigner.hun-ren.hu (J.L.); botzheim.lilla@wigner.hun-ren.hu (L.B.); 2 Rehabilitation Clinic, Semmelweis University, Szanatorium Street 19, 1121 Budapest, Hungary; feher.melinda@semmelweis.hu (M.F.); klaubera@t-email.hu (A.K.); 3 University of Pecs, Ifjujsag Street 6, 7624 Pecs, Hungary; 4 Faculty of Information Technology and Bionics, Pazmany Péter Catholic University, Prater Street 50/A, 1083 Budapest, Hungary

**Keywords:** blood pressure, functional electrical stimulation, FES cycling, heart rate, power output, spinal cord injury

## Abstract

**Background**: Globally, over 15 million people live with spinal cord injury (SCI), which often leads to permanent motor impairment. In these cases, functional electrical stimulation (FES) can generate muscle forces and active movements in affected body parts, enabling patients to perform cycling tasks using their own paralyzed legs. Incomplete spinal cord injuries are more prevalent than complete injuries and FES cycling can be performed in both cases. However, differences in its effects between the two groups remain to be further investigated. Our objective is to compare the effects of FES-assisted cycling on blood pressure, heart rate, and power output in patients with incomplete (iSCI) versus complete (cSCI) spinal cord injuries. We aim to provide comparative data currently missing from existing research. **Methods**: Thirty-two patients (20 iSCI, 12 cSCI), completed at least ten FES cycling training sessions. Each session consisted of 30 min of cycling on a MOTOmed Viva2 cycle-ergometer (Reck GMBH, Betzenweiler, Germany) equipped with a multi-channel electrical stimulator. The outcome measures were assessed in each session four times: before and after the session, and approximately at the 10th and 20th minutes. Statistical analyses compared mean arterial pressure (MAP), heart rate (HR), average and peak power output between the two groups. **Results**: Regarding within session differences, the iSCI group maintained significantly higher MAP than the cSCI group at all measurement points. HR patterns also differed significantly, showing higher values in the iSCI group at the second and third measurement points. On the course of the sessions in iSCI patients, average and peak power output increased significantly from the first to the tenth session. In cSCI patients the average power output was nearly constant throughout the ten sessions. **Conclusions**: Patients with iSCI and cSCI show different cardiovascular adaptations, and increasing FES cycling power output indicates that patients with incomplete injuries can achieve greater improvements even after ten training sessions.

## 1. Introduction

Spinal cord injury (SCI) can cause incomplete or complete loss of motor, sensory and autonomic function, leading to secondary complications. The severity of impairment is classified using the American Spinal Injury Association (ASIA) Impairment Scale [1], which evaluates preserved muscle activity and sensory functions. Complete SCI (cSCI) involves total loss of sensory and motor function below the injury level (ASIA A), while incomplete SCI (iSCI) indicates that some neural communication, movement or sensation is still present (ASIA B-C-D) [2,3]. SCI frequently results in complications such as hyperreflexia, spasticity, contractures, muscle atrophy, and increased risk of pressure ulcers. Additional secondary complications closely link to the ASIA category and injury level and may include neuropathic pain, neurogenic bladder and bowel dysfunction, reduced bone density, cardiovascular degradation and dysfunction and sexual impairment [4]. Physical deconditioning and orthostatic hypotension are the primary factors in maladaptive remodeling of the peripheral vasculature after SCI. Resting blood flow in the legs is typically 30–40% lower than in healthy individuals [5], leading to reduced circulating blood volume. These changes increase cardiac workload, particularly on the left ventricle, and elevate the risk of thrombosis.

Functional electrical stimulation (FES)-assisted cycling is an important rehabilitation method that induces controlled muscle contractions in the lower limbs. By activating the muscles’ pump function, it mitigates secondary complications and provides a unique therapeutic option for individuals with SCI. During FES cycling, electrical impulses are applied to elicit active muscle forces for performing a controlled and repetitive pedaling motion. FES leg cycling can target both muscular and cardiovascular systems [6,7]. In a 4-month follow-up study of a chronic cSCI patient training for the Cybathlon FES bike race, Fattal et al. [8] reported a slight increase in maximal mean arterial pressure (MAP) at the beginning of training sessions, through values remained within 106–133 mmHg. Maximum heart rate (HR) showed no significant change and consistently remained below 90 bpm throughout. Theisen’s results align with the following: in five cSCI patients, HR began at 76 ± 16 BPM, then slightly decreased in the first 2 min of the session, then increased to 90 ± 12 bpm and remained stable [9]. Similarly, Raymond et al. found in seven cSCI participants that HR initially decreased, then increased, while MAP slowly but steadily decreased during the session [10]. These research highlight that the cSCI population has decreased capability for operating on low MAP and HR adaptation in training through paralyzed muscles with FES cycling.

Although the iSCI group is highly heterogenous, ranging from minimal sensory function to near-normal motoric functioning, the literature should be interpreted according to each study’s specific aims. According to West et al., in chronic spinal cord injury, indices of cardiovascular function are more closely related to the degree of autonomic impairment than to the neurological completeness classification [5]. Saadeh et al.’s meta-analysis on acute and sub-acute SCI blood pressure (BP) indicates that post-injury BP stabilization is still not well understood [11].

FES cycling induces coordinated muscle contractions that contributes to muscle strengthening, motor relearning, spasticity management [12,13]—key components in the rehabilitation of SCI [14]. A systematic review provides further support for the association between improved aerobic fitness and increased power output through FES cycling training [7]. However, Duffell et al. followed 11 cSCI patients and found that maximal power output (PO) increased only in the first 25 weeks of FES tricycling, with no further improvements despite one year of training; peak PO remained under 30 W [15]. Mohr et al. [16] similarly reported maximal PO below 50 W after a comparable year-long program. Theisen measured under 15 W in 5 cSCI patients during a single FES session [9]. Griffin et al. observed modest improvements as well: in 18 chronic SCI participants (14 iSCI and 4 cSCI), PO increased from under 1 W up to 5 W after 10 weeks of training [17].

Duffel et al. [18] combined FES cycling with virtual reality in 11 chronic iSCI patients (ASIA B-C-D) over 12 sessions in 4 weeks. PO varied by ASIA class but did not exceed 30 W. Although tissue level limitations affect FES effectiveness in both cSCI and iSCI, the approach still improves overall function, measurable via PO, cardiac response or other indicators.

Although FES cycling has been widely studied, to our best knowledge, no prior research has examined such a large number of individuals using the same FES training protocol and compared the effect of FES cycling on heart rate and arterial pressure responses between homogeneous groups of people with complete and incomplete SCI. Our work complements the existing literature on the subacute phase of SCI. By performing session-by-session assessments, we highlight cardiovascular adaptations that occur over a short duration, rather than following time-intensive FES cycling training protocols.

The present study investigates the effects of FES cycling in iSCI and cSCI patients, evaluating MAP, HR, and PO to assess acute cardiovascular responses and rehabilitation outcomes. We hypothesized:

1. FES cycling induces significantly different responses when comparing groups with complete and incomplete spinal cord injuries in terms of MAP, HR, and performance metrics.
2. Even after just ten training sessions, the FES cycling performance of patients in the incomplete SCI group can be significantly improved, whereas this is not sufficient for improvement in patients with complete SCI.

For this retrospective study, the dataset was collected over several years [19,20,21,22,23] and organized in a consistent and comparable manner to allow reliable comparison and longitudinal interpretation. The study population represents subacute SCI patients who are undergoing low-effort FES cycling as a therapeutic intervention aimed at improving circulation and preserving muscle mass and nerve excitability.

## 2. Materials and Methods

### 2.1. Participants

A total of 32 patients were involved in this study. Data were assessed from 20 iSCI, defined with ASIA B and C classification, and 12 cSCI, defined as ASIA A (Table 1). Fourteen participants of the iSCI group have an ASIA B classification, meaning that their injury is sensory incomplete. They have preserved sensory function below the neurological level but have lost motor function, so they are unable to voluntarily move the pedal of the cycle ergometer. Participants with an ASIA C classification have more than half of the key muscles below the injury level with a muscle grade of less than 3. The cause of SCI varies between patients, with most being traumatic, few inflammatory and few due to stenosis of the spinal canal.

Additional inclusion criteria included stable mental and physiological condition, the ability to sit in a wheelchair during the day, the ability to participate in sports therapy at the clinic, and no cardiovascular disease that would have affected their participation in FES training.

The Ethics Committee of the National Institute for Medical Rehabilitation, Budapest, Hungary (presently Semmelweis University, Rehabilitation Clinic) provided approval for this research, and all patients gave written informed consent (approval number 20/2017/10/04).

### 2.2. Training

Each patient received FES cycling training twice a week, in total at least 10 training sessions. Our study included the results of the first ten training sessions for each subject. In the training sessions, a MOTOmed Viva 2 cycle-ergometer (Reck GMBH, Betzenweiler, Germany) was used. It was connected to an 8-channel commercial electrical stimulator (Rehamove, HASOMED GmbH, Magdeburg, Germany) or to a self-developed stimulator (Pázmány Péter Catholic University, Budapest, Hungary). The patient sat in their wheelchair and the cycle ergometer was positioned in front of the wheelchair at a distance that allowed unobstructed rotation of the pedal and appropriate knee extension. Bipolar surface electrodes (PG473W TENS ELEC 45 × 80 mm, FIAB, Firenze, Italy) were placed above the quadriceps and hamstrings muscles on both legs. Electrical impulses were delivered from the stimulator to the electrodes via wires. The impulse width was 300–350 µs and the frequency was 30–40 Hz. The timing of the stimulation based on the ergometer’s crank direction. The stimulator was connected to a rotation sensor that recorded the crank direction (100 Hz sampling frequency). The muscle activation pattern, as function of crank direction, was defined based on the muscle activity of healthy individuals [24].

Each training session lasted 30 min and consisted of a 5 min warm-up, 20 min of active cycling and a final 5 min cool-down. The motor moves the legs passively during the warm-up and cool-down phases, with no electrical stimulation. During the active stimulation phase, the current amplitude was increased until the patient’s muscles could generate cycling independently from the motor as a result of external muscle stimulation. The increase in current amplitude was dependent on the daily muscle condition of the participants. Patients’ muscle tone could vary from day to day (becoming more or less spastic), which means that the maximal current amplitude was reached where it did not achieve tetanic contraction but was sufficient to allow cycling with FES independently from the assistance of the motor for 20 min. This current amplitude varied across participants. The average maximal current intensity was 78 mA for patients with AIS A, 61 mA for AIS B and 59 mA for AIS C.

### 2.3. Assessments

For monitoring blood pressure (BP) and heart rate (HR), we applied automatic sphygmomanometer (OMRON HEALTHCARE EUROPE, Hoofddorp, the Netherlands).

The BP measuring protocols have changed slightly over the years in terms of the exact time of the measurements. Hence, BP data were adjusted based on the timing of intrasession measurement. Practically HR and BP were assessed at four time points: before and after each session, and intermediate values were aligned and presented as representative values at approximately one-third and two-thirds time of the session’s duration. The second time point fell between 7 and 14 min, and the third between 20 and 23 min. These are represented in the manuscript as the 10th and 20th minute marks, respectively.

#### Evaluated Parameters

- Mean arterial pressure (MAP) were calculated for better informativeness and clarity [mmHg] from the BP values;
- Heart rate (HR) values [bpm];
- Modified maximal heart rate (maxHR) values for SCI population [bpm] [25];
- Average and peak power output indicated by the MOTOmed cycle-ergometer (W);
- The percentage of work done by the left leg indicated by the MOTOmed cycle-ergometer [%].

The (MAP) values were calculated by the following equation:

*MAP* = *dyas* + (*sys* − *d*ias)/3 (1)

MAP values, represented by a single number, indicate changes in systolic (sys) and diastolic (dias) values. This number, along with heart rate values, allows for a non-invasive characterization of the cardiovascular system’s response to our FES cycling training [26].

Modified maximal heart rate (maxHR) for participants was calculated by Zbogar kind of equation [25]:

*maxHR* = 208 − 0.7 *× age* (2)

We investigated how the highest HR values recorded during each training session relate to the maxHR for the respective age. The highest HR for each session and person was divided by the maxHR. Thus, we obtained a ratio for each session and person separately (maxHR ratio). Then, we averaged these ratios across people and analyzed them for 10 training sessions in both groups.

### 2.4. Statistics

For statistical evaluation, the normality of the data was investigated. Since the data did not show a normal distribution (checked by a Shapiro–Wilk test), non-parametric statistical tests were used.

We were interested in assessing and comparing the level of cardiovascular adaptation in the two groups. MAP and HR were recorded four times during one training session, and these values were averaged across patients and across the ten training sessions. Thus, for both groups, we obtained 2 curves based on the measurement points, whose shape and magnitude provide us with relevant information. The measured values were compared pairwise between the two groups, using a non-parametric independent T-test (Mann–Whitney U test) with Bonferroni correction.

Then, we compared the average and peak power output of the first, fifth and tenth training sessions within each group using Friedman’s test with Conover post hoc test. In addition, pairwise comparisons were made between the first, fifth and tenth training sessions between the two groups using a non-parametric independent two-sample T-test (Mann–Whitney U test) with Bonferroni correction.

## 3. Results

### 3.1. Mean Arterial Pressure and Heart Rate Within the Training Sessions

The mean and standard deviation (SD) of MAP and HR were compared between the cSCI and iSCI groups during the FES cycling training, summarizing the data from the first 10 training sessions (Table 2). In the comparison of the two groups, the MAP values were significantly different throughout the whole session, whereas the HR showed only significantly different during the active cycling phase of the session.

The MAP values showed similar trajectories between the two groups, but compared to patients with complete injury, patients with iSCI had significantly higher MAP at all measurement time points, with a moderate effect (before: *p* < 0.001, rb = 0.35; 10. min: *p* < 0.001, rb = 0.43; 20. min: *p* < 0.001, rb = 0.38; after: *p* < 0.001, rb = 0.29).

**Figure 1:**
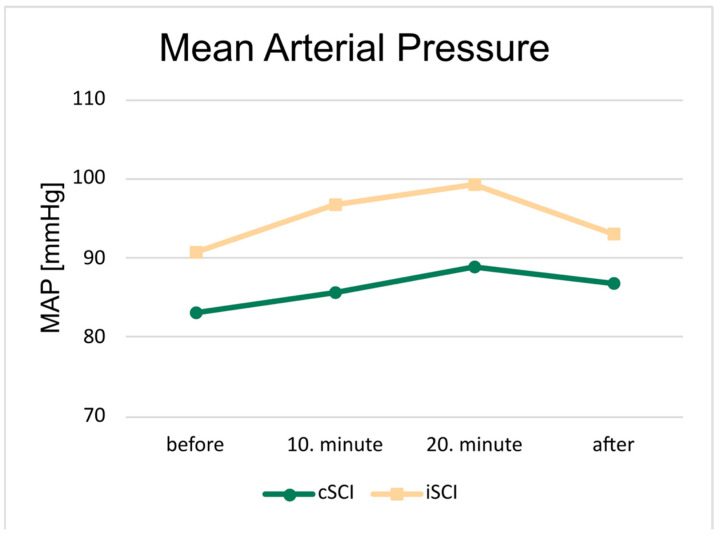
Mean arterial pressure (MAP) averaged across participants and training sessions separately for the groups with complete (cSCI) and incomplete (iSCI) spinal cord injuries (green and orange symbols respectively). Each marker represents a measurement point during a training session (circle: cSCI, square: iSCI). The MAP values of the iSCI groups were significantly higher in all measurement points.

The MAP values were higher at the 10th and 20th minute measurement points compared to the pre-training “before” value, which represents the resting value of the session, in both groups (Figure 1). The mean MAP values in the cSCI group ranged between 83 and 89 mmHg, while in the iSCI group, mean MAP values ranged from 90 to 100 mmHg.

**Figure 2:**
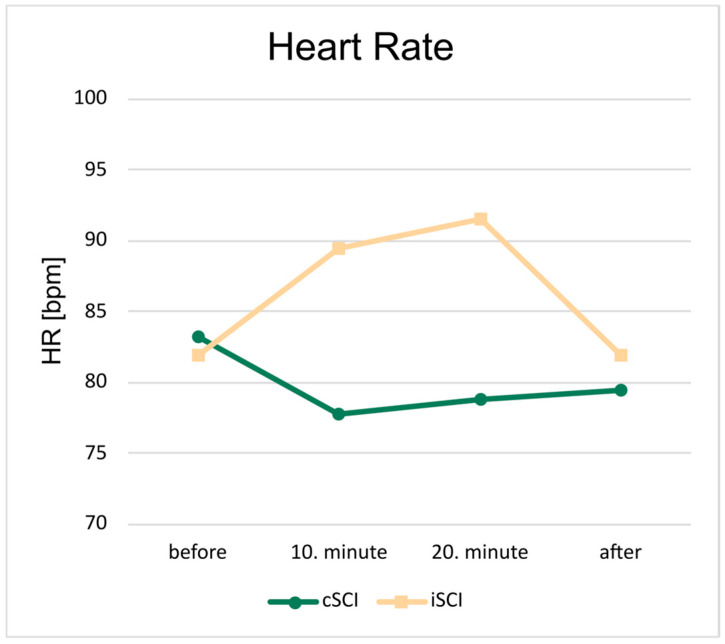
HR values averaged across patients and training sessions. Each marker represents a measurement point during a training session. The green line is the cSCI group, and the orange line is the iSCI group. The HR values of the iSCI groups were significantly higher in the 10th and 20th minutes of the training session. In addition, the two-line graphs demonstrate distinct patterns: one shows an initial increase followed by a decrease, whereas the other decreases at first before increasing later in the session.

In the four measurement points, the HR values were also measured (Figure 2). The trajectory and the range of HR values differed between the two groups. In patients with cSCI, the HR decreased until the 10th minute of the training session and then it increased slightly. Notably, the final HR value was lower than the pre-training (before) value. In iSCI cases, the pre-training HR was slightly lower than in the cSCI group and steadily increased until the third measurement point before sharply decreasing by the end of the training session. The HR values ranged between 77 and 83 bpm, for cSCI cases and between 81 and 92 bpm for incomplete cases. The cSCI patients started the training with their highest HR, while the iSCI persons reached their highest HR at around third measurement point. However, the trajectory was different; there were significant differences between the two groups in the second and third measurement points (in 10. min and in 20. min) with moderate effect size (10. min: *p* < 0.001, rb = 0.39; 20. min: *p* < 0.001, rb = 0.37).

### 3.2. Maximal Heart Rate on the Course of the Training Sessions

**Figure 3:**
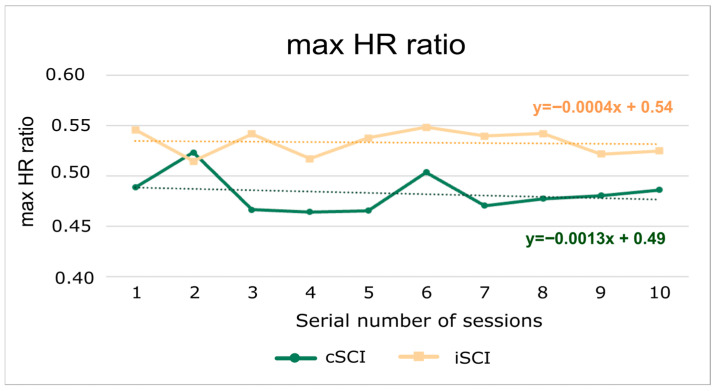
The maxHR ratios averaged across patients in 10 training sessions separately. The maxHR ratio means the ratio of the highest HR value recorded during the training session to the maximum HR value achievable for age. The dashed lines represent the linear regression for both groups across the 10 sessions.

Investigating the maxHR ratio on the course of the training sessions, we did not find any significant differences between the two groups or between training sessions. The values stagnate in the case of iSCI patients and show a slight decrease in the case of cSCI patients (Figure 3).

### 3.3. Power Output

The power output values (both average and peak power output) were averaged across participants in each group.

**Figure 4:**
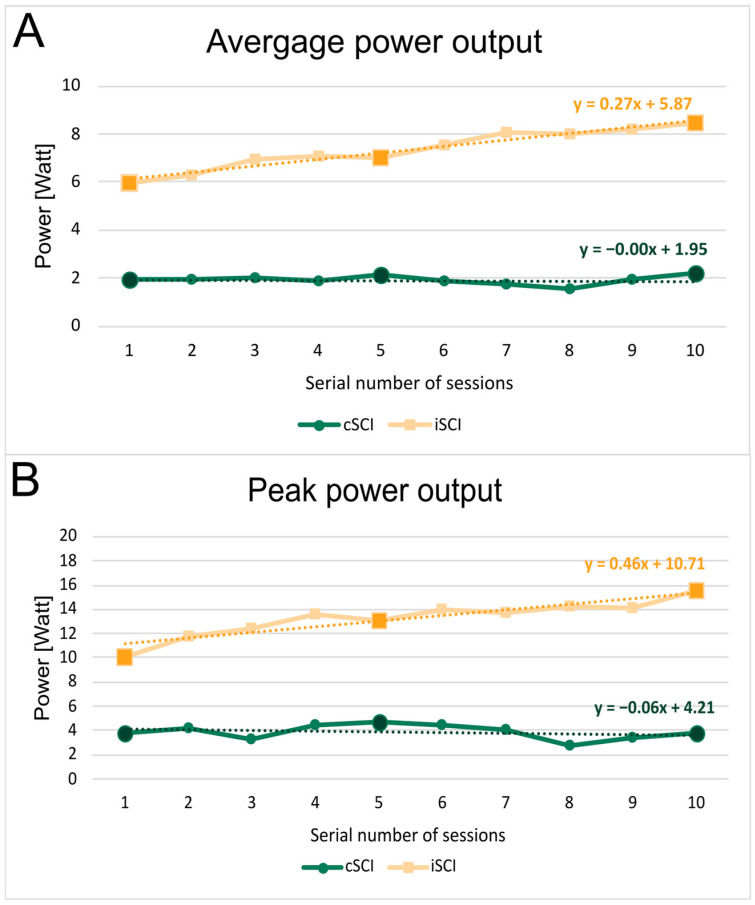
The average (**A**) and peak (**B**) power output achieved during the 10 training sessions, averaged across patients. The dashed lines represent the linear regression for both groups across the 10 sessions.

In the cSCI group, the average power output was nearly constant during the ten training sessions (Figure 4A. There was a slight increase in the peak power output in the fifth training session compared to the first, and then a slight decrease in the tenth training session (Figure 4B), but it remained greater than in the first session. In iSCI patients, both average and peak power output showed observable increases during the ten training sessions (Figure 4).

The average and peak power output were compared between the training sessions and between the groups (Table 3).

For intra-group statistics (based on Friedman test), no significant differences were found between the first, fifth and tenth training sessions in either average or peak power output within cSCI group. In contrast, significant differences were found between the first and tenth training sessions in average power output in the iSCI group with low-moderate effect size (*p* = 0.008, Kendall’s W = 0.18). Moreover, the peak power output was also significantly higher in the tenth session compared to the first (*p* < 0.001) and to the fifth (*p* = 0.029) with moderate effect size (Kendall’s W = 0.30), in this group (Table 3).

For the inter-group comparison (based on Mann–Whitney U test), at the beginning there were no significant differences between the two groups regarding power output. In the iSCI group, the average and peak power output were significantly higher in the tenth session than in the cSCI group with moderate-high effect size (average power output: *p* = 0.027, rb = 0.47; peak power output: *p* = 0.005, rb = 0.60). The highest average power output was 2.19 W for the cSCI and 8.47 W for the iSCI group. Similar difference was observed in the peak power output, the highest value for cSCI group was 4.75 W and 15.55 W for the iSCI group.

In the cSCI group, both average and peak power output remained nearly consistent during the 10 trainings sessions, indicating minimal variability. In contrast, the iSCI group exhibited a monotonic increase in power output across the 10 training sessions, with a statistically significant improvement observed by the 10th session compared to the first.

### 3.4. Symmetry in the Work Performed by the Two Legs

**Figure 5:**
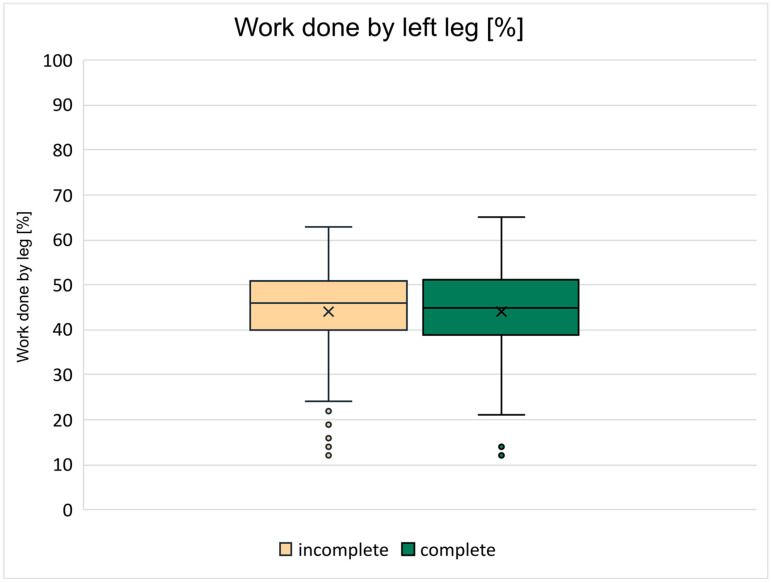
The boxplot diagram of the percentage of the work done by left leg, over the 10 training sessions and the patients. The median value of the cSCI group is 46, and the mean value is 44.16. The median value of the iSCI group is 45, and the mean value is 44.12.

The percentage of work done by the left leg is the percentage value displayed by the ergometer. Figure 5 shows the average value across training sessions and across patients separately for the two groups in boxplots. The most values were between 40 and 50% in both groups with a mean of 44.1% in both groups.

Our stimulation strategy was to ensure that symmetry was maintained and to get the two legs to perform as equally as possible. The average work done of 44–56% for left–right legs was considered acceptable based on our results.

## 4. Discussion

Our results indirectly indicate that there is a difference in the cardiovascular response and muscle strength gain between patients with complete and incomplete spinal cord injuries during low-intensity FES cycling training.

### 4.1. MAP and HR Values

With cSCI, circulation is more severely reduced in the areas below the injury than in incomplete injury [5,27] which highly influences how we can intervene in the cardiovascular system by stimulation of paralyzed muscles. We found significant differences in MAP values between the two groups during the active cycling phase, which may be related to the type of injury and the reduced circulation. According to De Groot et al. [27], blood flow in paralyzed limbs is reduced in patients with cSCI, but it can be assumed that this reduction is less severe in iSCI cases. This difference could explain why iSCI patients have a higher range in MAP and HR values. Our training indicated approximately 10 mmHg difference between minimal and maximal values of the averaged MAP and a 10 bpm difference in HR in iSCI patients, whereas these differences were approximately 5 mmHg and 5 bpm, respectively, in cSCI patients (Table 2). Although other studies examine the various cardiovascular effects of FES cycling, the ranges we obtained are similar to those described by Fattal et al., Kjaer et al. and Saadeh et al. [8,11,28].

Nobrega et al. examined the effect of active and passive cycling on cardiac parameters in able-bodied patients (Passive cycling was performed using a tandem bicycle where the crank revolution was provided by a second rider). They found that MAP values increased, and HR values remained constant for passive cycling. In contrast, MAP values remained constant and HR values increased for active cycling. These mean different control strategies for passive and active cycling modes. In our study, the MAP values increased for both groups during the training session, which is similar for passive cycling in able-bodied participants. Meanwhile, the HR values increased in the iSCI group, like in active cycling in Nobrega’s study. That suggests that cardiovascular control is limited in this disability, but the iSCI patients’ values seem closer to the able-bodied cases [24].

In our study, while the initial HR values are very similar in both groups, they increased in the iSCI groups as a result of FES cycling and decreased at the end of the training session. This trend corresponds to healthy cases [29]; moreover, if able-bodied participants are cycling even on low power output (around 25 Watts) their heart rate and systolic values increase a little more than 15 bpm and 10 mmHg while diastolic values practically does not change [30]. In the case of our participants the mean arterial pressure changed in average by 10 mmHg for the iSCI group and 5 mmHg for the cSCI group. Thus, the exercise does not induce big challenge to the heart but still affects the blood pressure. In contrast, in the cSCI group, a sharper decrease followed by a moderate increase in HR values can be observed as a result of the training. Similar response was also observed by Theisen and Raymond [9,10]. The initial decrease in HR may be due to a sudden increase in circulatory volume. The probable explanation for this phenomenon is that during FES cycling, the stagnant blood volume in the lower limbs is returned to circulation. In order to better interpret HR behavior in the cSCI group, it is important to distinguish between autonomic impairment and the effects of mechanical venous return. In cases of complete motor injury, the ability of the heart to appropriately adjust chronotropic and vascular responses to exercise is limited due to reduced supraspinal control over sympathetic cardiovascular pathways, similar to the impairment in bladder and bowel functions [31]. Therefore, HR modulation in cSCI is not solely a reflection of central autonomic control, but can also be strongly influenced by peripheral hemodynamic changes. During FES cycling, electrically induced muscle contractions act as a peripheral muscle pump, mechanically increasing venous return from paralyzed limbs. This sudden increase in preload can temporarily activate cardiopulmonary and arterial baroreceptors, leading to a reflex-mediated drop in HR before a delayed compensatory response arises.

General blood pressure strategies agree that the MAP value should be maintained above 85 mmHg, in order to maintain enough nutrition and O2 to whole body for SCI population. In our study, MAP values were higher than 85 mmHg through the whole sessions for iSCI groups. This suggests that the low intensity FES cycling, in conjunction with our HR values, could help in preventing blood pressure issues. It may be worthwhile to perform FES training for a longer period of time and at a higher intensity in complete cases to achieve similar effect as in iSCI [11].

The maxHR ratio does not change noticeably as a result of training in either group. Although the maxHR ratio stagnates in persons with iSCI, their power output still slightly improves. This suggests that this group was able to adapt to training without having to significantly increase their HR. In patients with cSCI, the maximum HR decreases slightly, while power output stagnates. This may indicate a weaker ability to adapt to increased physical load through paralyzed limbs.

These results support our hypothesis that from a cardiovascular perspective the two groups respond differently to the training. For those with complete injuries, active pedaling during FES cycling training may represent a more significant circulatory load, thus slowing down the adaptation process.

### 4.2. Power Output

Initially, there was no significant difference between the power output of the two groups. However, after 10 training sessions, a significant increase was observed in the average and peak power output of the iSCI group, while the power output of the cSCI group remained rather stagnant. The increase in power output indirectly indicates an increase in the patient’s endurance and muscle strength, as has been examined in several previous studies [7,15,16,32,33].

Although these studies also observed an increase in power output in the cSCI group, these were partly the results of already-trained patients and partly the results of training that had been carried out over several months. In the case of the patients presented in our study, we examined the values of the first 10 FES training sessions, so the measured values were significantly influenced by the patients’ ability to adapt.

It should be noted that the power output values we measured are lower than those generally seen in the literature. One of the main reasons for this is that our FES cycling protocol required a minimum cadence of 10 rpm; at slower speed muscles generate greater torque, which increases muscle strength more effectively [34].

The differences in power output observed between the two groups are consistent with the differences in cardiovascular values. Overall, our results indicate that FES cycling therapy started at an early stage in the group with iSCI results in significant improvement in both blood pressure and power output values after just a few sessions, indirectly improving the patients’ endurance (Nineteen of our twenty iSCI patients began the training in less than 6 months after their injury). At the same time, based on our results, a slower adaptation process can be observed in the group with cSCI. This can be observed not only during the training program, but also within a single training session. HR values suggest that active pedaling during FES cycling training may represent a more significant circulatory load for those with complete injuries, thereby slowing down the adaptation process.

The symmetry of the work done by the two legs did not show differences between the two groups. This indicates that electrode placement, current adjustment and ergonomic adjustments for patients were sufficient to maintain a similar workload distribution in both groups.

Including a short training duration, and some variability between therapists, our study highlights the value of early FES intervention across SCI populations, with a customized approach based on injury completeness. The consistent maxHR ratios across sessions show that training is safe for the cardiovascular system, even early after injury.

These results support both hypotheses proposed:

1. There are significant group-level differences in cardiovascular and performance outcomes during FES cycling between incomplete and complete SCI patients.
2. Even short-term training (10 sessions) results in measurable performance gains for iSCI patients, whereas cSCI cases require longer or more intense protocols to reach improvements.

### 4.3. Limitations

Since the data on which our retrospective study is based were collected over many years, some limitations were encountered during the evaluation of the data collected in the training sessions. The variety of training leaders introduced some subjectivity; for instance, the adjustment of the current amplitude may have varied depending on the person conducting the session. Furthermore, HR and MAP measurements occur only approximately at the 10th and 20th minutes.

The 10 FES cycling training sessions were determined because it often takes several weeks for patients to achieve a physiologically stable condition appropriate for FES training. During the short inpatient rehabilitation period at the Clinic, only a limited time remains for actual FES cycling when the patient is able to independently transfer to the training facility.

There is indeed a significant difference in motor function between patients classified as ASIA B and ASIA C: while individuals with an ASIA B classification have no voluntary motor function below the level of injury, those classified as ASIA C retain some degree of motor function, although more than half of the key muscles below the injury level have a muscle grade of less than three. This distinction is clinically important, as it reflects a meaningful difference in functional potential and responsiveness to rehabilitation interventions such as FES cycling training.

The time since injury is also an important factor that should be considered. Our patient groups were relatively homogeneous, as most of the patients were in the subacute phase. Only three of the thirty-three patients had their injury more than 2 years before starting the training. We did not make comparisons based on the time since injury; future multi-site studies with larger cohorts would be needed to achieve a more detailed patient classification.

## 5. Conclusions

The above-mentioned findings demonstrate that FES cycling training is significantly increased the power output (even if it still means low power output values) for patients with iSCI, even within a short intervention period and using low-intensity training protocols, whereas patients with cSCI showed more moderate adaptability, with limited improvements in power output and cardiovascular response. These differences reflect physiological inequalities, such as reduced autonomic regulation. We acknowledge that these values do not indicate a strong training effect on the cardiac aspect, but they provide insight into the physiological state of SCI patients in the subacute phase.

To make FES cycling more effective in SCI rehabilitation, the literature has already demonstrated ways to achieve this, though these have been tested on heterogeneous groups. Progressively loaded FES cycling has been shown to increase trabecular bone density and muscle cross-sectional area when performed frequently and over a long period of time [35]. Furthermore, hybrid exercise modalities that involve the upper and lower limbs driven by FES elicit a greater aerobic demand and oxygen uptake than exercises that only use the arms, thereby improving cardiovascular effectiveness [36] Based on these findings, we recommend implementing different training protocols according to injury type (complete or incomplete). For iSCI patients, we recommend introducing FES cycling early in the rehabilitation phase, even with low-intensity protocols, to preserve muscle mass and maintain physiological stability. By contrast, cSCI patients should undergo more intensive protocols involving longer active cycling sessions and progressive resistance to achieve clinically relevant improvements in cardiovascular parameters (MAP and HR) and muscular performance (peak oxygen uptake) within an appropriate timeframe [35,36,37]. The low power output values observed in our cohort can primarily be attributed to the patients’ subacute status and limited prior involvement in FES cycling. Since the measurements reflect the first ten training sessions, performance was strongly influenced by the patients’ early neuromuscular adaptation and their need to become adapted to electrically induced cycling. In the subacute phase, the declining muscle mass, and incomplete cardiovascular adaptation also limit power generation. Therefore, the measured power output should be interpreted in the context of early-stage rehabilitation and the patients’ initial adaptation to FES cycling, rather than as an indicator of limited therapeutic effectiveness.

We believe that our current study can contribute to the widespread and more effective clinical application of FES cycling therapies.

## Figures and Tables

**Table 1:** Description of patients, age, gender: male (M) and female (F); ASIA Impairment Scale: A: no motor and sensation below the injury B: no motor but some sensation below the level of injury C: some motor and some sensation below the level of injury; number of patients according to neurological injury level: cervical (C), thoracic (T), lumbar (L); number of patients according to time since injury.

	Total	cSCI	iSCI
Patients/group	*n* = 32	*n* = 12	*n* = 20
Age (years)	41.7 ± 13.57	38.0 ± 10.27	43.81 ± 14.96
Sex (M/F)	24/8	9/3	15/5
ASIA grade (A/B/C)	12/14/6	12/0/0	0/14/6
Injury level (C/T/L)	7/20/5	1/10/1	6/10/4
Time since injury (<6 months/6 months–2 years/2 years<)	25/4/3	6/3/3	19/1/0

**Table 2:** The descriptive statistics of the mean arterial pressure (MAP) and heart rate (HR) and statistical comparison of the measurement points between the complete an incomplete spinal cord injured group. Only training sessions with data available for all four measurement points were included in the statistical analysis (in the Supplementary Materials). The significance level was *p* < 0.05 (bold in the table).

**Mean arterial pressure during FES cycling**								
	complete (cSCI)				incomplete (iSCI)			
	before	10. min	20. min	after	before	10. min	20. min	after
Mean [mmHg]	83.1	85.6	88.8	86.8	90.7	96.8	99.2	93.1
SD [mmHg]	11.8	12.1	12.9	12.5	11.4	13.4	14.6	11.7
Comparison between groups								
	before		10. min		20. min		after	
*p* values	<0.001		<0.001		<0.001		<0.001	
**Heart rate during FES cycling**								
	complete (cSCI)				incomplete (iSCI)			
	before	10. min	20. min	after	before	10. min	20. min	after
Mean [bpm]	83.2	77.8	78.8	79.4	81.9	89.4	91.6	81.9
SD [bpm]	13.2	12.2	13.9	10.8	15.8	19.7	23.8	13.4
Comparison between groups								
	before		10. min		20. min		after	
*p* values	0.335		<0.001		<0.001		0.147	

**Table 3:** The descriptive statistics of the average and peak power output achieved during the 10 training sessions averaged across patients, as well as a comparison of values from sessions first, fifth, and tenth within each group and between the two groups. The significance level was *p* < 0.05 (significant difference are highlighted in bold in the table).

Average power output during active cycling						
	complete (cSCI)			incomplete (iSCI)		
	1. session	5. session	10. session	1. session	5. session	10. session
Mean [W]	1.93	2.13	2.19	5.94	7.05	8.47
SD [W]	1.59	1.68	1.54	7.06	8.84	8.82
Comparison between session within groups						
	1. session–5. session		5. session–10. session		1. session–10. session	
cSCI—*p* value	1.000		0.251		0.251	
iSCI—*p* value	0.266		0.098		**0.008**	
Comparison between groups						
	1. session: cSCI/iSCI		5. session: cSCI/iSCI		10. session: cSCI/iSCI	
*p* values	0.158		0.089		**0.027**	
Peak power output during active cycling						
	complete			incomplete		
	1. session	5. session	10. session	1. session	5. session	10. session
Mean [W]	3.75	4.75	3.83	10.10	13.05	15.55
SD [W]	3.47	3.86	2.37	11.82	14.42	13.65
Comparison between sessions within groups						
	1. session–5. session		5. session–10. session		1. session–10. session	
cSCI—*p* value	0.157		0.718		0.284	
iSCI—*p* value	0.096		**0.029**		**<0.001**	
Comparison between groups						
	1. session: cSCI/iSCI		5. session: cSCI/iSCI		10. session: cSCI/iSCI	
*p* values	0.159		0.123		**0.005**	

## Data Availability

Data is contained within the article or Supplementary Materials.

## Supplementary Materials

The following supporting information can be downloaded at: https://www.mdpi.com/article/10.3390/jcm15051967/s1.

## Abbreviations

The following abbreviations are used in this manuscript:

ASIA	American Spinal Injury Association
BP	Blood pressure
BPM	Beat per minute
cSCI	Complete spinal cord injury or injured
DIAS	Diastole
HR	Heart rate
iSCI	Incomplete spinal cord injury or injured
MAP	Mean arterial pressure
SCI	Spinal cord injury or injured
SYS	Systole

## References

[1] Rupp R., Biering-Sørensen F., Burns S.P., Graves D.E., Guest J., Jones L., Read M.S., Rodriguez G.M., Schuld C., Tansey K.E. (2021). International Standards for Neurological Classification of Spinal Cord Injury Revised 2019. Top. Spinal Cord Inj. Rehabil..

[2] Noonan V.K. (2023). A Look at Spinal Cord Injury in Canada: Rick Hansen Spinal Cord Injury Registry (RHSCIR)—2021 SCI Data Summary. Top. Spinal Cord Inj. Rehabil..

[3] Biering-Sørensen F., Kirshblum S.C. (2018). International Perspectives on Spinal Cord Injury Care. Spinal Cord Medicine.

[4] Guest J., Datta N., Jimsheleishvili G., Gater D.R. (2022). Pathophysiology, Classification and Comorbidities after Traumatic Spinal Cord Injury. J. Pers. Med..

[5] West C.R., Bellantoni A., Krassioukov A.V. (2013). Cardiovascular Function in Individuals with Incomplete Spinal Cord Injury: A Systematic Review. Top. Spinal Cord Inj. Rehabil..

[6] Fodor A., Naszlady M.B., Mravcsik M., Klauber A., Cserháti P., Laczko J., Horváth M. (2022). Effect of FES Controlled Cycling Training on Cardiovascular and Pulmonary Systems in a Spinal Cord Injured Patient. Curr. Dir. Biomed. Eng..

[7] van der Scheer J.W., Goosey-Tolfrey V.L., Valentino S.E., Davis G.M., Ho C.H. (2021). Functional Electrical Stimulation Cycling Exercise after Spinal Cord Injury: A Systematic Review of Health and Fitness-Related Outcomes. J. Neuroeng. Rehabil..

[8] Fattal C., Sijobert B., Daubigney A., Fachin-Martins E., Lucas B., Casillas J.M., Azevedo C. (2020). Training with FES-Assisted Cycling in a Subject with Spinal Cord Injury: Psychological, Physical and Physiological Considerations. J. Spinal Cord Med..

[9] Theisen D., Fornusek C., Raymond J., Davis G.M. (2002). External Power Output Changes during Prolonged Cycling with Electrical Stimulation. J. Rehabil. Med..

[10] Raymond J., Davis G.M., Van Der Plas M.N., Groeller H., Simcox S. (2000). Carotid Baroreflex Control of Heart Rate and Blood Pressure during ES Leg Cycling in Paraplegics. J. Appl. Physiol..

[11] Saadeh Y.S., Smith B.W., Joseph J.R., Jaffer S.Y., Buckingham M.J., Oppenlander M.E., Szerlip N.J., Park P. (2017). The Impact of Blood Pressure Management after Spinal Cord Injury: A Systematic Review of the Literature. Neurosurg. Focus.

[12] Dolbow D.R., Gorgey A.S., Johnston T.E., Bersch I. (2023). Electrical Stimulation Exercise for People with Spinal Cord Injury: A Healthcare Provider Perspective. J. Clin. Med..

[13] Alashram A.R., Annino G., Mercuri N.B. (2020). Changes in Spasticity Following Functional Electrical Stimulation Cycling in Patients with Spinal Cord Injury: A Systematic Review. J. Spinal Cord Med..

[14] Holtz K.A., Lipson R., Noonan V.K., Kwon B.K., Mills P.B. (2017). Prevalence and Effect of Problematic Spasticity After Traumatic Spinal Cord Injury. Arch. Phys. Med. Rehabil..

[15] Duffell L.D., Donaldson N.D.N., Perkins T.A., Rushton D.N., Hunt K.J., Kakebeeke T.H., Newham D.J. (2008). Long-Term Intensive Electrically Stimulated Cycling by Spinal Cord-Injured People: Effect on Muscle Properties and Their Relation to Power Output. Muscle Nerve.

[16] Mohr T., Andersen J.L., Biering-Sørensen F., Galbo H., Bangsbo J., Wagner A., Kjaer M. (1997). Long Term Adaptation to Electrically Induced Cycle Training in Severe Spinal Cord Injured Individuals. Spinal Cord.

[17] Griffin L., Decker M.J., Hwang J.Y., Wang B., Kitchen K., Ding Z., Ivy J.L. (2009). Functional Electrical Stimulation Cycling Improves Body Composition, Metabolic and Neural Factors in Persons with Spinal Cord Injury. J. Electromyogr. Kinesiol..

[18] Duffell L.D., Paddison S., Alahmary A.F., Donaldson N., Burridge J. (2019). The Effects of FES Cycling Combined with Virtual Reality Racing Biofeedback on Voluntary Function after Incomplete SCI: A Pilot Study. J. Neuroeng. Rehabil..

[19] Mravcsik M., Klauber A., Laczko J. FES Driven Cycling: Increased Crank Resistance in the Case of Lower Level of Injury—Comparison of Case Studies. Proceedings of the 21st Annual Meeting of the International Functional Electrical Stimulation Society.

[20] Fodor A., Váraljai L., Naszlady M., Mravcsik M. A funkcionális elektromos stimulációval végzett kerékpározási protokollok hatása a munkára [The effect of functional electrical stimulation cycling protocols on workload]. Proceedings of the XVIII National Sport Science Congress.

[21] Fodor A., Laczkó J., Botzheim L., Mravcsik M., Prószéky G., Szederkényi G. (2024). Cardiac Response of Patients with Complete and Incomplete Spinal Cord Injury to Functional Electrical Stimulation Driven Cycling Training. PhD Proceedings—Annual Issues of the Doctoral School, Faculty of Information Technology and Bionics, Pázmány Péter Catholic University.

[22] Ernyey D.M., Botzheim L., Mravcsik M., Laczko J., Horvath M. (2022). Funkcionális elektromos stimulációval szabályozott kerékpározás paraplégek számára. [Cycling controlled by functional electrical stimulation for paraplegics]. Fizioterápia.

[23] Katona P., Pilissy T., Tihanyi A., Laczko J. (2014). The Combined Effect of Cycling Cadence and Crank Resistance on Hamstrings and Quadriceps Muscle Activities during Cycling. Acta Physiol. Hung..

[24] Nobrega A.C., Williamson J.W., Friedman D.B., Araujo C.G., Mitchell J.H. (1994). Cardiovascular Responses to Active and Passive Cycling Movements. Med. Sci. Sports Exerc..

[25] Zbogar D., Eng J.J., Noble J.W., Miller W.C., Krassioukov A.V., Verrier M.C. (2017). Cardiovascular Stress During Inpatient Spinal Cord Injury Rehabilitation. Arch. Phys. Med. Rehabil..

[26] Nath Kundu R., Biswas S., Das M. (2017). Mean Arterial Pressure Classification: A Better Tool for Statistical Interpretation of Blood Pressure Related Risk Covariates. Cardiol. Angiol. Int. J..

[27] De Groot P.C.E., Van Kuppevelt D.H.J.M., Pons C., Snoek G., Van Der Woude L.H.V., Hopman M.T.E. (2003). Time Course of Arterial Vascular Adaptations to Inactivity and Paralyses in Humans. Med. Sci. Sports Exerc..

[28] Kjær M., Pott F., Mohr T., Linkis P., Tornøe P., Secher N.H. (1999). Heart Rate during Exercise with Leg Vascular Occlusion in Spinal Cord- Injured Humans. J. Appl. Physiol..

[29] Miyai N., Shiozaki M., Yabu M., Utsumi M., Morioka I., Miyashita K., Arita M. (2013). Increased Mean Arterial Pressure Response to Dynamic Exercise in Normotensive Subjects with Multiple Metabolic Risk Factors. Hypertens. Res..

[30] Miyai N., Arita M., Miyashita K., Morioka I., Shiraishi T., Nishio I. (2002). Blood Pressure Response to Heart Rate During Exercise Test and Risk of Future Hypertension. Hypertension.

[31] Hou S., Rabchevsky A.G. (2014). Autonomic Consequences of Spinal Cord Injury. Compr. Physiol..

[32] Kirshblum S., Snider B., Eren F., Guest J. (2021). Characterizing Natural Recovery after Traumatic Spinal Cord Injury. J. Neurotrauma.

[33] Fornusek C., Davis G.M. (2008). Cardiovascular and Metabolic Responses During Functional Electric Stimulation Cycling at Different Cadences. Arch. Phys. Med. Rehabil..

[34] Fornusek C., Davis G.M. (2004). Maximizing Muscle Force via Low-Cadence Functional Electrical Stimulation Cycling. J. Rehabil. Med..

[35] Frotzler A., Coupaud S., Perret C., Kakebeeke T.H., Hunt K.J., Donaldson N.d.N., Eser P. (2008). High-Volume FES-Cycling Partially Reverses Bone Loss in People with Chronic Spinal Cord Injury. Bone.

[36] Taylor J.A., Picard G., Widrick J.J. (2011). Aerobic Capacity With Hybrid FES Rowing in Spinal Cord Injury: Comparison With Arms-Only Exercise and Preliminary Findings With Regular Training. PM&R.

[37] Vestergaard M., Jensen K., Juul-Kristensen B. (2022). Hybrid High-Intensity Interval Training Using Functional Electrical Stimulation Leg Cycling and Arm Ski Ergometer for People with Spinal Cord Injuries: A Feasibility Study. Pilot Feasibility Stud..

